# Supplementary material for: Machine learning algorithms accurately identify free-living marine nematode species
Source: PeerJ. 2023 Oct 9;11:e16216. doi: 10.7717/peerj.16216 (PMC10569207; doi:10.7717/peerj.16216)
Supplement: Supplemental Information 1 — The green color indicates species that were excluded from the analysis due to poor taxonomical descriptions either by the absence of information of characters or were limited to a single specimen. [file peerj-11-16216-s001.docx]

| Table S1. List of valid *Acantholaimus* species based on Worms. The green color indicates species that were excluded from the analysis due to poor taxonomical descriptions either by the absence of information of characters or were limited to a single specimen. | |
| --- | --- |
| *A. aheneus* Bussau ,1993 | *A. macramphis* Gourbault & Vincx, 1985 |
| *A. akvavitus* Gerlach, Schrage & Riemann, 1979 | *A. maks Gerlach*, Schrage & Riemann, 1979 |
| *A. angustus* Bussau,1993 | *A. marliae Manoel*, Silva & Esteves, 2017 |
| *A. arminius* Gerlach, Schrage & Riemann, 1979 | *A. megamphis* Vivier, 1985 |
| *A.arthrochaeta* Miljutina & Miljutin, 2012 | *A. microdontus* Gourbault & Vincx, 1985 |
| *A. barbatus* Miljutina & Miljutin, 2012 | *A. minutus* (Vitiello, 1972) Gerlach, Schrage & Riemann, 1979 |
| *A.bidentatus* Esteves & Neres, 2022 | *A. obviatus* Vivier, 1985 |
| *A. caecus* Bussau, 1993 | *A. occultus* Bussau, 1993 |
| *A. calathus* Gerlach, Schrage & Riemann, 1979 | *A. polydentatus*Gerlach, 1951 |
| *A. cornutus* Miljutina & Miljutin, 2012 | *A. pugious* [Manoel, Esteves & Neres, 2022](https://nemys.ugent.be/aphia.php?p=taxdetails&id=1610689) |
| *A. coruscus* Bussau, 1993 | *A. quadridentatus* Jensen, 1985 |
| *A. cyathibucca* Vivier, 1985 | *A. quintus* Gerlach, Schrage & Riemann, 1979 |
| *A. elegans* Jensen, 1988 | *A. robustus* Miljutina & Miljutin, 2012 |
| *A.  formosus* Miljutina, Miljutin & Tchesunov, 2013 | *A. septimus* Gerlach, Schrage & Riemann, 1979 |
| *A. gathumai* Muthumbi & Vincx, 1997 | *A. setosus* Vitiello, 1970 |
| *A. geraerti* Muthumbi & Vincx, 1997 | *A. sieglerae* Miljutina & Miljutin, 2012 |
| *A. gigantasetosus* Vivier, 1985 | *A. skukinae* Miljutina, Miljutin & Tchesunov, 2013 |
| *A. heipi* Muthumbi & Vincx, 1997 | *A. spinicauda* (Vitiello, 1970) Gerlach, Schrage & Riemann, 1979 |
| *A. incomptus* Vivier, 1985 | *A. tchesunovi* Miljutina & Miljutin, 2012 |
| *A. invaginatum* Muthumbi & Vincx, 1997 | *A. tectus* Bussau, 1993 |
| *A. iubilus* Gerlach, Schrage & Riemann, 1979 | *A.  veitkoehlerae*Miljutina & Miljutin, 2012 |
| *A. longisetosus*Allgén, 1933 | *A. vermeuleni* Muthumbi & Vincx, 1997 |
| *A. longistriatus* Gourbault & Vincx, 1985 | *A. verscheldi* Muthumbi & Vincx, 1997 |

***Table of Supplementary Materials***
