# Supplementary material for: Machine learning algorithms accurately identify free-living marine nematode species
Source: PeerJ. 2023 Oct 9;11:e16216. doi: 10.7717/peerj.16216 (PMC10569207; doi:10.7717/peerj.16216)
Supplement: Supplemental Information 2 — The green color indicates species that were excluded from the analysis due to poor taxonomical descriptions either by the absence of information of characters or were limited to a single specimen. [file peerj-11-16216-s002.docx]

| *Table of Supplementary Materials* | | |
| --- | --- | --- |
| Table S2. List of valid *Sabatieria* species based on Worms. The green color indicates species that were excluded from the analysis due to poor taxonomical descriptions either by the absence of information of characters or were limited to a single specimen. | | |
| *S. abyssalis* (Filipjev, 1918) Filipjev, 1922 | *S. finitima* Fadeeva & Belogurov,1984 | *S. paracupida* Wieser & Hopper, 1967 |
| *S. alata* Warwick, 1973 | *S. flecha* Pastor de Ward, 2003 | *S. paradoxa* Wieser & Hopper, 1967 |
| *S. ancudiana* Wieser, 1954 | *S. foetida* Gagarin & Thanh, 2008 | *S. parapraedatrix* Leduc ,2013 |
| *S. arctica* (Allgén, 1954) | *S. furcillata* Wieser,1954 | *S. paraspiculata* Botelho et al, 2007 |
| *S. arcuata* Wieser ,1954 | *S. granulosa* Vitiello & Boucher, 1971  S. granifer Wieser, 1954 | *S. paravulgaria* Filipjev, 1946  *S. paramacramphis* Leduc & Zhao, 2023 |
| *S. armata* Gerlach, 1952 | *S. heipi* Chen & Vincx ,2000 | S.parvamphis Yang,Guo, Chen & Lin, 2019 |
| *S. articulata* Fu&Leduc, 2019 | *S. heterospiculum* Allgen,1953 | *S. parvula* Gagarin & Thanh, 2006 |
| *S. aspera* Sergeeva, 1973 | *S. heterura* (Cobb,1898) Filipjev, 1918 | *S. pellucida* Allgen,1959 |
| *S. australis* Allgén, 1929 | *S. intacta* Fadeeva & Belogurov, 1984 | *S. pisinna* Vitiello ,1970 |
| *S. balbutiens* Leduc, 2013 | *S. intermissa* Wieser, 1954 | *S. pomarei* (Boucher,1973) |
| *S. bathycopia* Leduc, 2013 | *S. kelletti* Platt, 1983 | *S. praebosporica* Sergeeva,1973 |
| *S. bitumen* Botelho et al. 2007 | *S. kolaensis* (Ssaweljev,1912) Filipjev, 1922 | *S. praedatrix* De Man, 1907 |
| *S. bubulba* Leduc, 2013 | *S. labium* Botelho, Esteves & Fonseca-Genevois 2014 | *S. propisinna* Vitiello, 1976 |
| *S. celtica* Southern,1914 | *S. lawsi* Platt, 1983 | *S. pulchra (*Schneider, 1906) Riemann,1970 |
| *S. cettensis* Rouville, 1903 | *S. lepida* (Vitiello,1976) | *S. pumila* Leduc ,2013 |
| *S. challengerensis*, Leduc,2013 | *S. longicaudata* Filipjev, 1922 | *S. punctata (*Kreis, 1924) |
| *S. chukchensis* Yang, Guo, Chen & Lin, 2019 | *S. longisetosa* (Kreis,1929) | *S. rota* Gerlach, 1957 |
| *S. cleopatris* Micoletzky, 1924 | *S. longispinosa* Lorenzen, 1971 | *S. rotundicauda* Allgen,1959 |
| *S. conicauda* Vitiello, 1970 | *S. lucia Muthumbi* ,1997 | *S. sanjosensis* Pastor de Ward, 2003 |
| *S. conicoseta*, Guo, Chang & Yang, 2018 | *S. lyonessa* Warwick, 1977 | *S. sarcina* Vitiello, 1976 |
| *S.coomansi* Chen&Vincx, 1999 | *S. maboyae* Gourbault & Vincx, 1990 | *S. sinica* Zhai, H.; Wang, C.; Huang, Y, 2019 |
| *S. curvispiculata* Gagarin, 2013 | S*. macramphis* Lorenzen,1971 | *S. spiculata* Botelho, Da Silva, Esteves & Fonseca-Genevois, 2007 |
| *S. demani* Shuurmans Stekhoven, 1935 | *S. major* Yang, Guo, Chen & Lin, 2019 | *S. splendens* (Hopper, 1967) |
| *S. dispunctata* Rosli,Leduc & Probert, 2014 | *S. mawsoni* (Cobb, 1914) Wieser,1954 | *S. stekhoveni* Vitiello, 1970 |
| *S. doancanhi*, Tu, Thanh, Smol & Vanreusel, 2008 | *S. megadena* Leduc 2017 | *S. stenocephalus* Huang & Zhang,2006 |
| *S. dodecaspapillata* (Kreis, 1929) | *S. microsetosa* Timm, 1967 | *S. strigosa* Lorenzen, 1971 |
| *S. dorylaimopsoides* Allgén, 1959 | *S. migrans* Jensen & Gerlach, 1977 | *S. subrotundicauda* Botelho, Da Silva, Esteves & Fonseca-Genevois, 2007 |
| *S. effilata Shuurmans* Stekhoven,1950 | *S. mortenseni* (Ditlevsen, 1921) Filipjev, 1922 | *S. supplicans* Gerlach, 1956 |
| *S. elongata* Jayasree & Warwick,1977 | *S. multisupplementia* Yang, Guo, Chen & Lin, 2019 | *S. taboguillensis* (Allgen,1947) Wieser,1954 |
| *S. execulta* Leduc, 2013 | *S. norwegica* Allgen,1931 | *S. tenuiseta* Allgen, 1959 |
| *S. exilis* Botelho et al. 2009 | *S. ornata* (Ditlevsen, 1918) Filipjev, 1922 | *S. triplex* Wieser, 1954 |
| *S. falcifera* Wieser,1954 | *S. pacifica* Allgén, 1947 | *S. tubilaima* Allgen,1959 |
| *S. fidelis* Botelho et al, 2009 | *S. palmaris* Fadeeva & Belogurov, 1984 | *S. vasicola* Vitiello ,1970 |
| *S. filicauda* Allgen,1951 | *S. parabyssalis* Wieser, 1954 | *S. verteris* Botelho, Esteves & Fonseca-Genevois, 2014 |
| [S. paramacramphis Leduc & Zhao, 2023](https://nemys.ugent.be/aphia.php?p=taxdetails&id=1649638) | *S. paracupida* Wieser & Hopper, 1967 | *S. wieseri* Platt,1985 |

Table S2 (continued)
