# Supplementary material for: Machine learning algorithms accurately identify free-living marine nematode species
Source: PeerJ. 2023 Oct 9;11:e16216. doi: 10.7717/peerj.16216 (PMC10569207; doi:10.7717/peerj.16216)
Supplement: Supplemental Information 3 — The number of individuals required for carrying out the classification of Acantholaimus species. [file peerj-11-16216-s003.docx]

| Table S3: The number of individuals required for carrying out the classification of *Acantholaimus* species. | | |
| --- | --- | --- |
| Species | Number of individuals from the descriptions | Number of individuals used for validation |
| *A. akvavitus* | 3 | 0 |
| *A. angustus* | 11 | 2 |
| *A. arminius* | 2 | 0 |
| *A. arthrochaeta* | 7 | 1 |
| *A. barbatus* | 10 | 1 |
| *A. caecus* | 2 | 0 |
| *A. cornutus* | 4 | 1 |
| *A. coruscus* | 2 | 0 |
| *A. elegans* | 2 | 0 |
| *A. formosus* | 3 | 0 |
| *A. gathumai* | 2 | 0 |
| *A. geraerti* | 2 | 0 |
| *A. gigantasetosus* | 3 | 0 |
| *A. heipi* | 2 | 0 |
| *A. incomptus* | 2 | 0 |
| *A. invaginatum* | 2 | 0 |
| *A. iubilus* | 3 | 0 |
| *A. longistriatus* | 3 | 0 |
| *A. macramphis* | 2 | 0 |
| *A. maks* | 3 | 0 |
| *A. marliae* | 2 | 0 |
| *A. megamphis* | 3 | 1 |
| *A. microdontus* | 5 | 1 |
| *A. minutus* | 3 | 0 |
| *A. obviatus* | 2 | 0 |
| *A. occultus* | 3 | 0 |
| *A. polydentatus* | 2 | 0 |
| *A. quadridentatus* | 2 | 0 |
| *A. quintus* | 4 | 1 |
| *A. robustus* | 6 | 1 |
| *A. septimus* | 4 | 1 |
| *A. setosus* | 2 | 0 |
| *A. sieglerae* | 5 | 1 |
| *A. skukinae* | 3 | 0 |
| *A. spinicauda* | 3 | 0 |
| *A. tchesunovi* | 3 | 0 |
| *A. tectus* | 2 | 0 |
| *A. veitkoehlerae* | 12 | 2 |
| *A. vermeuleni* | 2 | 0 |
| *A.verscheldi* | 7 | 1 |

***Table of Supplementary Materials***
