# Supplementary material for: Machine learning algorithms accurately identify free-living marine nematode species
Source: PeerJ. 2023 Oct 9;11:e16216. doi: 10.7717/peerj.16216 (PMC10569207; doi:10.7717/peerj.16216)
Supplement: Supplemental Information 4 — The number of individuals required for carrying out the classification of Sabatieria species. [file peerj-11-16216-s004.docx]

| ***Table of Supplementary Materials*** | | |
| --- | --- | --- |
| Table S4: The number of individuals required for carrying out the classification of *Sabatieria* species | | |
| Species | Number of individuals from the descriptions | Number of individuals used for validation |
| *S. alata* | 4 | 1 |
| *S. ancudiana* | 2 | 0 |
| *S. armata* | 5 | 1 |
| *S. balbutiens* | 4 | 1 |
| *S. bitumen* | 2 | 0 |
| *S. bubulba* | 3 | 0 |
| *S. celtica* | 11 | 2 |
| *S. conicauda* | 5 | 1 |
| *S. conicoseta* | 10 | 1 |
| *S. demani* | 2 | 0 |
| *S. dispunctata* | 2 | 0 |
| *S. doancanhi* | 2 | 0 |
| *S. dorylaimopsoides* | 3 | 0 |
| *S. elongata* | 4 | 1 |
| *S. execulta* | 5 | 1 |
| *S. exilis* | 3 | 0 |
| *S. falcifera* | 2 | 0 |
| *S. fidelis* | 4 | 1 |
| *S. flecha* | 2 | 0 |
| *S. furcillata* | 2 | 0 |
| *S. granifer* | 10 | 2 |
| *S. heipi* | 2 | 0 |
| *S. heterura* | 2 | 0 |
| *S. intermissa* | 2 | 0 |
| *S. kelleti* | 2 | 0 |
| *S. labium* | 3 | 0 |
| *S. lawsi* | 2 | 0 |
| *S. lepida* | 10 | 2 |
| *S. longicaudata* | 6 | 1 |
| *S. longispinosa* | 7 | 1 |
| *S. lucia* | 2 | 0 |
| *S. lyonessa* | 2 | 0 |
| *S. macramphis* | 2 | 0 |
| *S. major* | 3 | 0 |
| *S. mawsoni* | 2 | 0 |
| *S. migrans* | 2 | 0 |
| *S. mortenseni* | 3 | 0 |
| *S. multisupplementia* | 3 | 1 |
| *S. ornata* | 17 | 2 |
| *S. parabyssalis* | 4 | 1 |
| *S. parapraedatrix* | 5 | 1 |
| *S. paraspiculata* | 2 | 0 |
| *S. paravulgaria* | 2 | 0 |
| *S. parvamphis* | 3 | 0 |
| *S. pisinna* | 4 | 1 |
| *S. propisinna* | 6 | 1 |
| *S. pulchra* | 20 | 2 |
| *S. punctata* | 11 | 2 |
| *S. sanjosensis* | 2 | 0 |
| *S. sinica* | 5 | 1 |
| *S. spiculata* | 2 | 0 |
| *S. stekhoveni* | 6 | 1 |
| *S. stenocephalus* | 6 | 1 |
| *S. strigosa* | 3 | 0 |
| *S. subrotundicauda* | 2 | 0 |
| *S. triplex* | 2 | 0 |
| *S. vasicola* | 4 | 1 |
| *S.chukchensis* | 3 | 0 |
| *S.pomarei* | 6 | 1 |
| *S.praedatrix* | 3 | 1 |
